# Sex specificity of dispersal behaviour and flight morphology varies among tree hollow beetle species

**DOI:** 10.1186/s40462-022-00340-7

**Published:** 2022-09-24

**Authors:** Sandra Martínez-Pérez, Eduardo Galante, Estefanía Micó

**Affiliations:** Instituto de Investigación CIBIO (Centro Iberoamericano de la Biodiversidad), Universidad de Alicante, 03690 San Vicente del Raspeig, Alicante Spain

**Keywords:** Saproxylic beetles, Sex biased dispersal, Primary sex ratio, Flight performance, Dispersal behaviour, Wing loading, Wing aspect ratio

## Abstract

**Background:**

Flight performance and dispersal behaviour can differ between sexes, resulting in sex-biased dispersal. The primary sex ratio of populations may also explain dispersal bias between sexes, as this bias may evolve with the primary sex ratio to reduce intrasexual competition. Although dispersal bias between sexes is relevant to population dynamics, there are few studies on sex-biased dispersal in insects. We studied the flight performance and dispersal behaviour of seven saproxylic beetle species associated with tree hollows from a sex perspective. We also analysed the possible coevolution of flight performance with the primary sex ratio.

**Methods:**

Wing loading and wing aspect ratio were used as measures of the flight performance of species and sexes. Dispersal behaviour was explored by analysing the frequency of each sex in interception traps *versus* the primary sex ratio obtained by tree hollow emergence traps using contingency tables and posthoc standardized residuals. A more active flight behaviour was expected for the sex with higher capture frequency in the interception traps. To explore the causes of flight performance bias between sexes, we searched for possible correlations between wing loading or wing aspect ratio and primary sex ratio using Pearson’s correlation coefficient.

**Results:**

Wing loading and wing aspect ratio differed between species and sexes, with flight performance being higher in males than in females for four of the seven species analysed. Dispersal behaviour and flight performance matched in the case of *Elater ferrugineus*; males showed higher flight performance and were the most collected sex in the interception traps (more active flyers). In contrast, the higher flight activity of *Cetonia carthami aurataeformis* females was not correlated with a higher flight performance than that of males. Moreover, we found that a bias in the primary sex ratio towards females is often correlated with a decrease in female flight performance.

**Conclusions:**

We stress that flight performance and dispersal behaviour of sexes do not always go hand in hand. Moreover, the relationship between the sex ratio and flight performance bias between sexes is not driven by competition within the most abundant sex. The inclusion of a sex perspective in insect dispersal studies would be useful to detect dispersal bias between sexes and its causes and would allow for further analysis of its effects on population dynamics.

## Background

Approaching insect dispersal studies from a sex perspective is informative since the insect dispersal patterns often exhibit large differences at the sex level, affecting population dynamics [1, 2]. Studies on dispersal bias between sexes are common in mammals and birds; however, dispersal ability and dispersal behaviour remain underexplored in insects, and few studies have been approached from a sex perspective [3–5].

Species dispersal traits may differ between females and males due to morphological, physiological and behavioural differences [6, 7]. Therefore, when individuals of one sex disperse more than another, sex-biased dispersal (SBD) occurs [7]. Different theoretical hypotheses have been proposed to explain SBD behaviour: the “resource competition hypothesis” (LRC) [8], “local mate competition hypothesis” (LMC) [9] and the “inbreeding avoidance hypothesis” (IAH) [10]. However, the most widely accepted hypothesis to explain SBD is the LRC, which explains why the interaction between competition for local resources and competition for local mates drives population SBD [7, 8]. A higher dispersal rate may evolve in the most abundant sex to reduce intrasexual competition in the natal patch [5, 8, 11, 12]. Selective pressures may therefore act differently on the sexes [2], leading to sexual dimorphism in dispersal behaviour [7, 8, 13–15].

Understanding dispersal bias between sexes in insects and what factors may affect them can help to predict the vulnerability of communities, as dispersal bias has an effect on population dynamics and species’ response to environmental changes [3, 7]. Males can contribute to the genetic rescue of populations, but cannot contribute to demographic rescue as females do [12]. Therefore, populations with male-biased dispersal may be at greater risk of extinction than those with a female bias [12].

One of the most interesting communities for studying insect dispersal behaviour within forest ecosystems are the saproxylic assemblages that inhabit tree hollows.^1^ First, tree hollows are considered a keystone microhabitat for European forest biodiversity conservation [16–18]. Second, cavity availability and spatial connectivity are currently jeopardized by several factors, such as forest fragmentation, climatic change, forestry and the abandonment of cultural practices such as tree pollarding (note that tree pollarding accelerates the formation of tree cavities) [16, 19, 20]. Third, fauna that inhabit stable habitats, such as tree hollow microhabitats, are likely to have a lower dispersal capacity than those that inhabit other more unstable habitats [1, 21, 22]. Finally, they include endangered species that are obligate cavity inhabitants [23]. Thus, in the current context of global insect decline [24], it is of particular interest to study the forces that explain the dispersal of such vulnerable assemblages.

Dispersal studies can be approached by direct measurements in the field as a mark–release–recapture method; however, this method presents serious difficulties for investigating individuals who travel long distances [25] and for species whose field observations are hard to evaluate; this is especially true for rare, saproxylic species, whose small populations sizes or peculiar habitat (i.e., species that develop in microhabitats such as tree hollows) make them difficult to detect in the field [26, 27]. In these cases, indirect measurements based on flight morphology, such as wing loading (WL) (body mass divided by wing area) and wing aspect ratio (AR)(wing length divided by wing width), can be used as a measure of species flight performance that can help detect possible differences in the success that species, or sexes, might have in colonizing new habitats. Although flight morphology has been questioned as a good indicator of species dispersal [28, 29], several studies have shown that traits such as WL could explain flight performance and a higher propensity to disperse, which indicates that flight morphology and flight performance would be correlated [1, 22, 30–32].

We assessed flight morphology traits and their possible coevolution with primary sex ratio of 7 beetle species associated with tree hollows in Mediterranean *Quercus* forests using different trapping methods (emergence *versus* interception traps) to determine the primary sex ratio and species dispersal behaviour [33]. Morphological dispersal traits such as WL and AR allowed us to analyse potential differences in flight performance between species and sexes. Moreover, we used the primary sex ratio, morphological traits (WL and AR) and the frequency of capture of each sex in the interception traps *versus* the primary sex ratio to analyse the possible causes of SBD and test for possible coevolution of SBD and the primary sex ratio.

We expected to find differences in flight performance between species and sexes, but these differences are not necessarily related to intraspecific competition [5, 33]. Furthermore, flight performance and dispersal behaviour of species and sexes do not necessarily go hand in hand, as when the benefits of dispersal overcome the physiological costs, dispersal of individuals will increase [12]. Finally, finding of differences in flight performance between sexes, would show the need to include a sex perspective in insect dispersal studies.

## Methods

### Study area

**Fig. 1:**
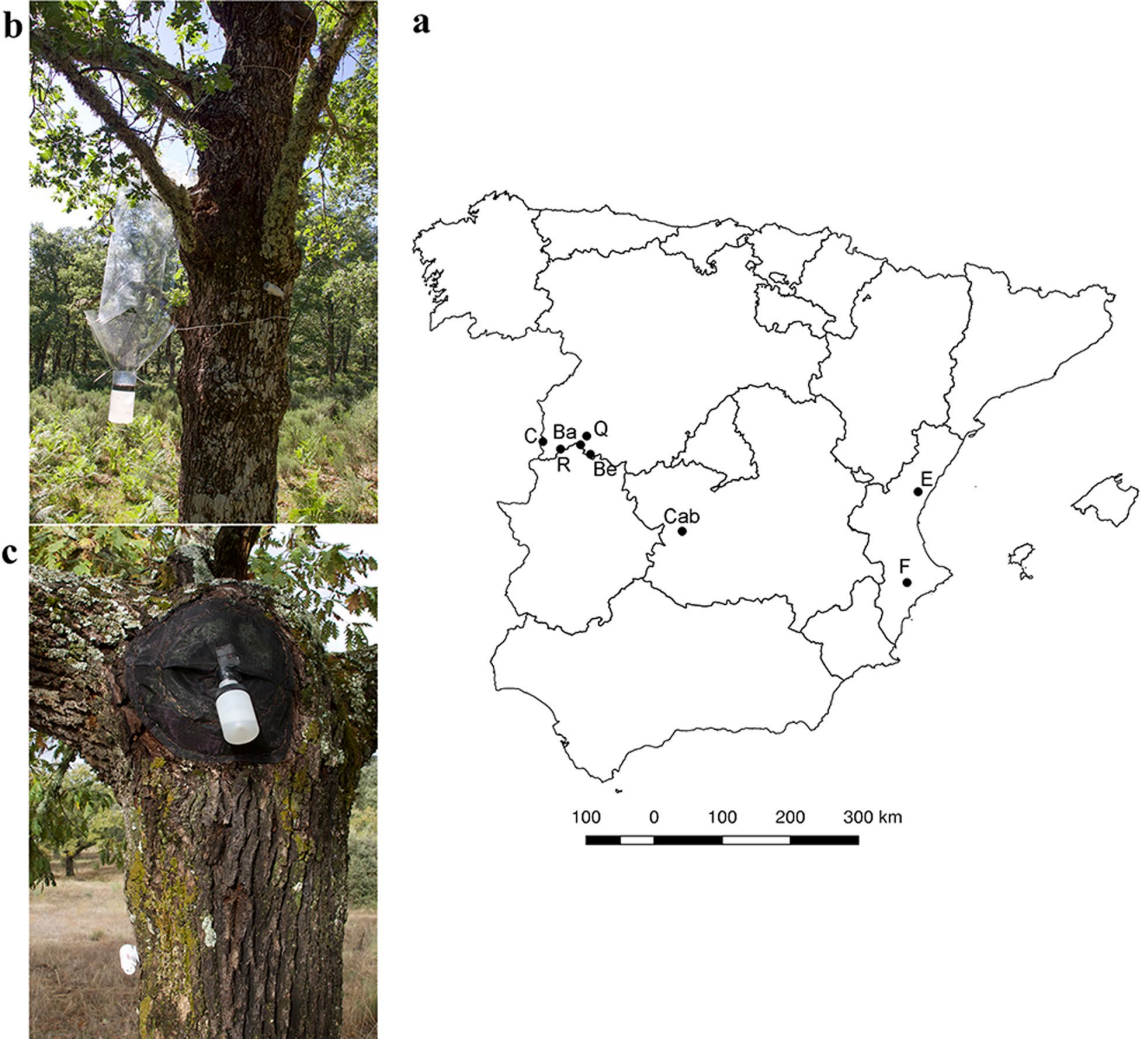
Study areas and trap types. **a** Map showing the distribution of the protected areas in the Iberian Peninsula sampled in the study. C: Biological Reserve ‘Campanarios de Azaba’ (Salamanca), R: El Rebollar Natural Area (Salamanca), Ba: Las Batuecas-Sierra de Francia Natural Park (Salamanca), Q: Sierra de las Quilamas Natural Area (Salamanca), Be: Sierra de Béjar UNESCO Biosphere Reserve (Salamanca), Cab: Cabañeros National Park (Ciudad Real), E: Sierra Espadán Natural Park (Castellón) and F: Font Roja Natural Park (Alicante). All the studied areas are characterised by mature forests of *Quercus* species **b** Window trap (WT) photo; **c** Hollow emergence trap (HET) photo

The data used in this study were collected from Mediterranean forests located in 8 protected areas of the Iberian Peninsula: the biological Reserve ‘Campanarios de Azaba’ (Salamanca), Sierra de las Quilamas Natural Area (Salamanca), El Rebollar Natural Area (Salamanca), Las Batuecas-Sierra de Francia Natural Park (Salamanca), Sierra de Béjar UNESCO Biosphere Reserve (Salamanca), Cabañeros National Park (Ciudad Real), Sierra Espadán Natural Park (Castellón) and Font Roja Natural Park (Alicante) (Fig. 1a, Table 1). All the study areas were characterized by mature forests of *Quercus* species.

**Table 1:** Number of traps and dominant tree species in each studied area

Site	Tree species sampled	Number HET	Number WT	Coordinates	Sampling year
Campanarios	*Quercus rotundifolia* Lam.	18	15	40° 29.769 N 6° 47.551 W	2010–2011
	*Quercus pyrenaica* Willd.	10	12		
	*Quercus faginea* Lam.	3	2		
	*Quercus suber *L.	0	1		
Quilamas	*Q. pyrenaica*	33	38	40° 35.642 N 6° 03.201 W	2012–2013 2014–2015
Rebollar	*Q. pyrenaica*	18	39	40°21.10 N 6°35.05 W	2014–2015 2017–2018
Cabañeros	*Q. suber*	9	37	39° 23.47 N 4° 29.14 W	2004–2005 2015–2016
	*Q. rotundifolia*	32	18		
	*Q. pyrenaica*	22	14		
	*Fraxinus angustifolia* Vahl.	27	13		
	*Q. faginea*	8	8		
Batuecas	*Q. rotundifolia*	30	45	40° 27.291 N 6° 08.088 W	2012–2013
Espadán	*Q. suber*	9	9	39° 52.00 N 0°17.30O	2015–2016
Font Roja	*Q. rotundifolia*	9	9	38°38.51 N 0° 32.46 W	2015–2016
Béjar	*Q. pyrenaica*	0	12	40°25.26 N 5°47.16O	2017–2018
Total		228	272		

Campanarios (Biological Reserve Campanarios de Azaba), Quilamas (Quilamas Natural Area), Rebollar (The Rebollar Natural Area), Cabañeros (Cabañeros National Park), Batuecas (Las Batuecas-Sierra de Francia Natural Park), Espadán (Sierra Espadan Natural Park), and Béjar (Sierra de Béjar UNESCO Biosphere Reserve)

### Beetle sampling and species selection

Beetles were sampled using 228 window traps (WTs) and 272 hollow emergence traps (HETs) (Fig. 1b, c, Table 1) which were placed in the tree species listed in Table 1. Large trees, with a diameter at breast height (DBH) greater than 20 cm, were selected for trap installation. Both trap types were present at all sites and were active for a complete year at each site (Table 1). Each WT consisted of two convergent transparent sheets (73 cm long and 42 cm wide) lying over a funnel and a collection container with preserving liquid (propylene or ethylene glycol) [34, 35] (Fig. 1b). Traps were hung on tree branches 1.5–2 m above the ground. These traps are effective for collecting saproxylic flying beetles associated with different types of tree microhabitats such as bark, tree hollows, dead branches or dead wood in the surroundings of the tree [36–38]. Each HET consisted of a black acrylic mesh that was completely sealed to the tree hollow through staples and a receptacle with preserving liquid (ethylene or propyleneglycol) attached to the mesh [39, 40] (Fig. 1c). This trap type is an effective method to collects species linked to tree hollows shortly after their emergence from immature stages, whether they are flightless or flying species [35]. Thus, we considered that HETs provide accurate information about populations’ primary sex ratio (calculated as females/males) (Table 2) [33]. In contrast, a high abundance of captures of one sex with respect to the other in the interception traps may provide information on the dispersal behaviour of each sex.

**Table 2:** Species observed frequencies of females and males and sex ratio in different sampling methods

Species	HET			WT		
	♂	♀	Primary sex ratio	♂	♀	Sex ratio
*Cetonia carthami aurataeformis*	130	234	1.8	6	134	22.2
*Protaetia cuprea*	12	118	9.8	8	210	26.5
*Protaetia mirifica*	4	14	3.5	0	6	–
*Cerambyx wellensii*	37	47	1.3	62	37	0.6
*Stictoleptura trisignata*	27	18	0.7	8	6	0.8
*Elater ferrugineus*	39	47	1.2	59	19	0.3
*Ischnodes sanguinicollis*	17	30	1.8	33	17	0.5

Sex ratio was calculated as females/males

Field studies were carried out from 2004 to 2018, and the samples were collected once a month during one complete year at each sampling site (Table 1).

We selected 7 saproxylic species belonging to 3 beetle families (Coleoptera: Cetoniidae, Elateridae and Cerambycidae) based on their presence in tree hollows, their IUCN Red List category of threat and their functional relevance [41]. All the species selected were represented by at least 20 individuals to allow for statistical comparisons. The selected species distribution, biology and IUCN Red List category are summarized in Table 3.

**Table 3:** Information about the distribution, biology and Red List category of threat of the species surveyed

Species	Distribution	Biology remarks	IUCN Red List category
*Cetonia carthami aurataeformis* Curti 1913 (Scarabaeidae: Cetoniidae)	Endemic subspecies of the Iberian Peninsula [42]	Larvae feed on wood and litter in tree hollows [43], while adults are flowers and fruits visitors [44]. This species has been considered an obligate species of tree hollows [33] and an ecosystem engineer^a^ of these peculiar microhabitats in Mediterranean forests [46]	–
*Protaetia (Potosia) cuprea* (Fabricius, 1775) (Cetoniidae)	Paleartic species [44]	Larvae have been considered facultative inhabitants of tree hollows while adults are flowers and fruits visitors [33, 44]	–
*Protaetia (Eupotosia) mirifica* (Mulsant, 1842) (Cetoniidae)	Rare species with Mediterranean distribution [47]	Larvae are obligate inhabitants of tree hollows [33]. Adults are often attracted by sugary resources	Vulnerable [23, 48, 49]
*Elater ferrugineus* Linnaeus, 1758 (Elateridae)	Western Palearctic species [50]	Larvae and adults are obligate inhabitants of tree hollows. Adults are predators of other saproxylic insects, and larvae can additionally feed on wood mould in cavities [51, 52]. This species has been considered a good indicator of tree hollows beetle diversity [53]	Near threatened [23]
*Ischnodes sanguinicollis* (Panzer, 1793) (Elateridae)	Palearctic species [50]	This species is an obligate inhabitant of tree hollows*.* Larvae and adults are predators [39, 54]	Vulnerable [23]
*Cerambyx welensii* (Kuster, 1846) (Cerambycidae)	Palearctic species [55]	They are considered ecosystem engineers^a^ [45, 56].The larvae are strictly xylophagous, while adults feed mainly on tree exudates or do not feed [57]	Near threatened [23, 49]
*Stictoleptura trisignata* (Fairmaire, 1852) (Cerambycidae)	Endemic species of the Iberian Peninsula [58]	The larvae of this species are considered xylophagous in different species of *Quercus* [58], while adults are flower visitors [59]	Near threatened [60]

^a^Are species that provide resources for other species because their activity alters the microhabitat conditions favouring other species fitness[45]

The sexes of each species were distinguished by differences in external morphological characters or by analysing the external genitalia when needed.

### Flight morphology

**Fig. 2:**
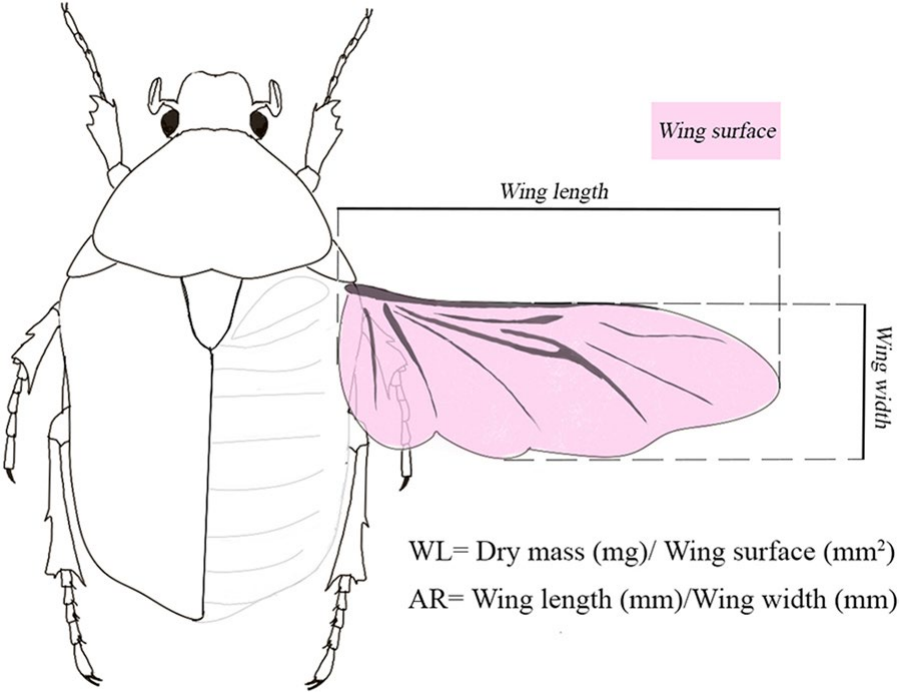
Schematic representation of the morphological traits measured associated with flight performance. WL: Wing loading; AR: Wing aspect ratio

The flight performance of the selected species selected and their sexes was inferred by indirect measurements of flight morphology, WL and AR [1, 22, 30]. Individuals were dried for 72 h at 30 °C in a drying oven and weighed on an AS82/220.R2 precision scale (RADWAG, Poland) with ± 0.01 mg accuracy (no individuals weighed less than 1 mg). Samples were rehydrated, and then the left membranous wing was removed and placed with transparent liquid glue on a slide under a cover slip. We measured wing surface, maximum wing length and wing width for 20 individuals (10 females and 10 males) of each species (Fig. 2) using a Leica M205C stereo microscope and Leica Application Suite software version 4.8. WL was calculated by dividing dry mass by wing surface, while AR was calculated by dividing maximum wing length by wing width [22, 61]. A low WL value represents flight that is more energetically efficient and has been interpreted as conferring better flight performance [22, 62]. Conversely, a high AR is indicative of higher wing movement speed [1, 22, 63], which implies that species with the highest AR may be more likely to colonize more habitats and cover longer distances than those with a low AR [64].

### Data analysis

Data from all sampling sites were combined to obtain a sufficient number of individuals for statistical analysis. In our case, all selected sites are located in protected areas characterized by mature forest (with large old trees > 20 cm DBH). The intraspecific variability in flight morphology can be affected by landscape structure (i.e., woodlands versus agricultural landscape), forest maturity [29, 33, 65, 66] and food resource availability [67]. Therefore, in this study, no intraspecific differences in flight-related morphology were expected between sites (all included in protected areas), as the landscape structure, forest maturity and conservation are not expected to differ much between sites. To analyse the differences in WL values and ARs between species and sexes, we first tested whether the data were normally distributed with the Shapiro–Wilk normality test [68]. We compared WL values and ARs between species with a Kruskal–Wallis test with multiple comparisons. When significant, we used a posthoc pairwise Wilcoxon test to identify differences between species, and the alpha value was adjusted following Bonferroni correction [69]. Comparisons between both sexes for each species were performed with a nonparametric Mann–Whitney U test for independent samples for WL measurements, except for *P. cuprea* and *P. mirifica,* for which we used a parametric Student’s t test. For AR trait comparisons between sexes, we used a parametric Student’s t test.

To test the contribution of sex ratio to the species dispersal behaviour, we analysed the frequency of capture of each sex in hollow emergence traps (HETs) with respect to that in interception traps (WTs). For this aim, we used 2 × 2 contingency tables based on a likelihood ratio χ^2^ test [70]. Standardized residuals were analysed to determine whether the observed frequency differed significantly from what would be expected by chance [71, 72].

Moreover, a pairwise correlation was calculated to evaluate the relationships between the primary sex ratio of each species and morphological flight traits by species and sex (female wing loading: WL_F; male wing loading: WL_M; female wing aspect ratio: AR_F; and male wing aspect ratio: AR_M). For this analysis, we calculated the Pearson rank correlation coefficients and their p values. We considered it as serious collinearity for pairwise correlation where *ρ* ≥ 0.75 [73].

## Results

### Flight morphology of species and sexes

**Fig. 3:**
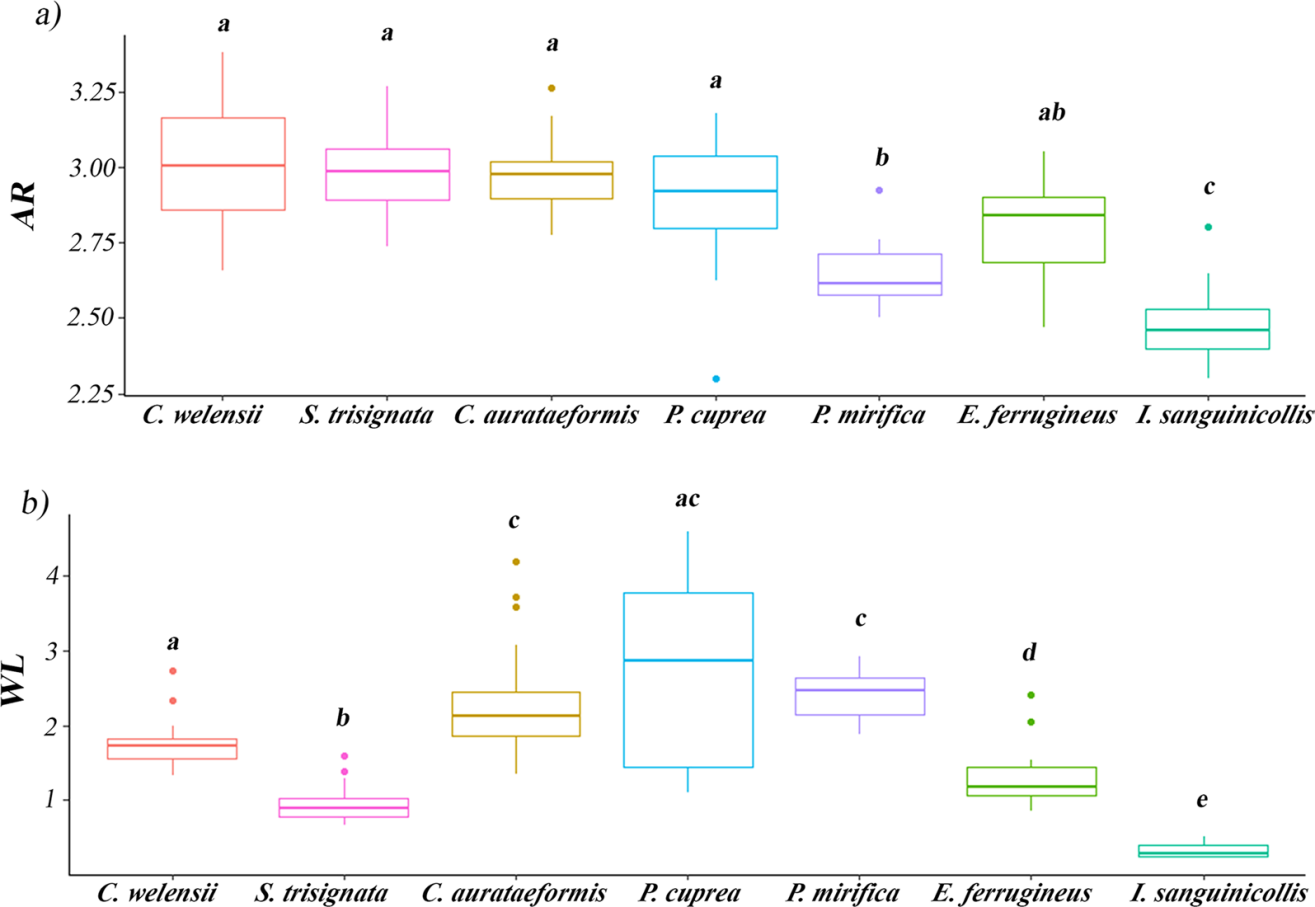
Boxplots showing **a** wing aspect ratio (AR) and **b** wing loading (WL) value for each species. Minimum whiskers, interquartile range boxes (first quartile (Q1), median, third quartile (Q3)), maximum whiskers and outlier symbols are plotted. Bars with different letters mark significant differences in the pairwise Wilcoxon test after Bonferroni correction (P < 0.008)

Morphological dispersal traits (WL and AR) showed differences among species (Kruskal–Wallis chi-squared test = 109.6, df = 6, p value < 2.2e-16) (Fig. 3). In general, AR varied less between species than WL.

*I. sanguinicollis* had the lowest AR, and *P. mirifica* and *E. ferrugineus* also showed a low AR (Fig. 3a). In contrast, WL differed among all species, except for Cetoniidae species (Fig. 3b). *I. sanguinicollis* and *S. trisignata* showed the lowest WL. In contrast, Cetoniidae species showed the highest WL (worst flight efficiency compared with the rest) (Fig. 3b).

**Fig. 4:**
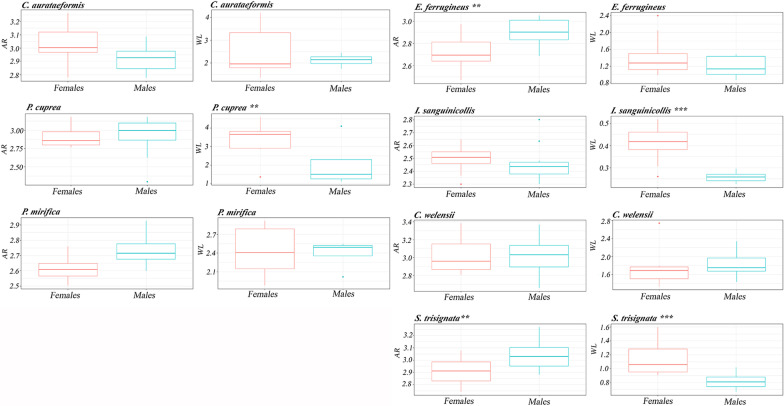
Boxplots showing wing aspect ratio (AR) and wing loading (WL) by species and sex. Minimum whiskers, interquartile range boxes (first quartile (Q1), median, third quartile (Q3)), maximum whiskers and outlier symbols are plotted. P = significance level by Mann–Whitney U test for WL analyses (with the exception of *P. cuprea* and *P. mirifica,* where we used a parametric t test) and P = significance level by parametric t test for AR analyses

Differences in flight performance between sexes were found within some species (Fig. 4). *E. ferrugineus* and *S. trisignata* presented a lower AR in females than in males. Similarly, *S. trisignata*, *P. cuprea* and *I. sanguinicollis* also showed significantly higher WL in females (Fig. 4).

### Dispersal behaviour

The results showed significant differences in the capture frequency of females and males of *C. c. aurataeformis* and *E. ferrugineus* for each type of trap (Table 4). However, the differences varied among them. Females of *C. c. aurataeformis* were captured more often than expected by chance in the interception traps (WTs) (indicating that the sex was more active in flight) than in the hollow emergence traps (HETs) (reflecting the primary sex ratio). In contrast, males of *E. ferrugineus* were captured more often than expected by chance in WTs (Table 4).

**Table 4:** Pearson’s χ ^2^ significance from the 2 × 2 contingency table test and post hoc standardized residuals

Species	Pearson’s χ^2^	Df	Prob. level	Standardized residuals post-hoc (Z critical value)		
				Sex	HET	WT
*Cetonia carthami aurataeformis*	50.05	1	**= 0.0000**	Females	**1.07**	**2.97**
				Males	**2.62**	**− 4.74**
*Protaetia cuprea*	3.67	1	**0.0311**	Females	− 0.41	0.31
				Males	1.65	− 1.27
*Protaetia mirifica*	1.6	1	0.5271^a^	–	–	–
*Cerambyx welensii*	5.5905	1	**0.01806**	Females	1.36	− 1.25
				Males	− 1.25	1.15
*Stictoleptura trisignata*	4.291e-31	1	1	–	–	–
*Elater ferrugineus*	14.373	1	**0.0001499**	Females	**2.10**	**− 2.21**
				Males	**− 1.72**	**1.81**
*Ischnodes sanguinicollis*	7.4782	1	**0.006245**	Females	1.51	**− **1.46
				Males	**− **1.46	1.42

Significant values in bold

^a^Yate’s correction was applied to the χ ^2^ test when the expected frequency value was less than 5

### Species sex ratio and flight performance bias between sexes

**Fig. 5:**
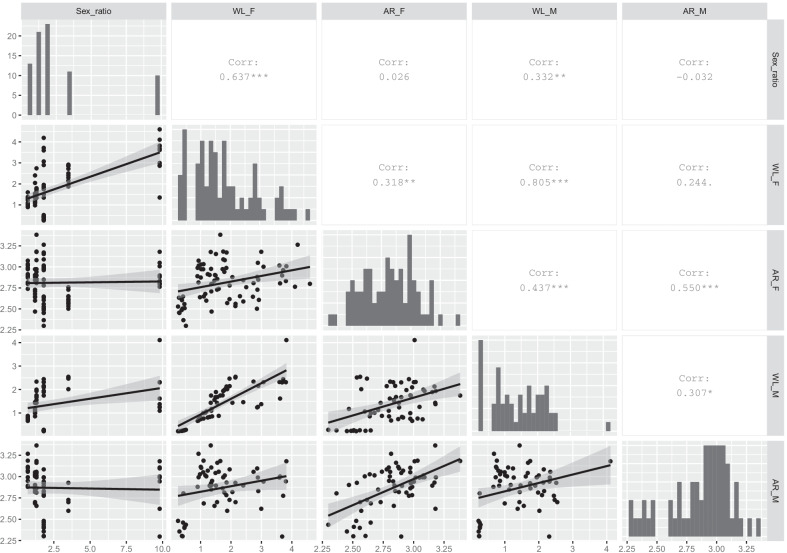
Pearson pair correlation plot between the primary sex ratio and morphological dispersal traits by sex (WL_F: female wing loading; WL_M: male wing loading; AR_F: female wing aspect ratio and AR_M: male wing aspect ratio). Primary sex ratio values levels near and below 1 are not represented in the Figure (*E. ferrugineus* and *S. trisignata*) but were included in the correlation. The cell number show the Pearson rank correlation coefficient (*ρ*) and p = significance level of the correlation, p < 0.05*, p < 0.005**, p < 0.0005***

The results of Pearson correlation coefficients showed moderate collinearity between the primary sex ratio and WL_F, while high collinearity (*ρ* ≥ 0.75) was shown between WL_F and WL_M. According to our results, an increase in the sex ratio biased towards females seems to be linked with an increase in WL_F (Fig. 5).

## Discussion

Our results showed that the inclusion of a sex perspective in insect dispersal studies could help not only to better understand the dispersal behaviour of some saproxylic beetle species inhabiting tree hollows but also to detect flight performance bias between sexes (key to population dynamics) and its possible causes. Additionally, our results suggest that intrasexual competition is not always a consequence of an imbalance in the sex ratio of populations. For the same reason a dispersal bias in favour of the more abundant sex according to the primary sex ratio cannot be assumed.

### Flight performance and dispersal behaviour

Morphological traits (WL and AR) were used to assess the flight performance of rare species that are poorly detectable with direct measurements in the field. Although the use of WL and AR as a proxy to measure the dispersal of insect species has been questioned [28, 29], the study of flight-related traits has been extensively studied in some insects orders such as Lepidoptera, Trichoptera or Hemiptera, where the results could explain the higher migratory success of species or sexes [26, 31, 32]. In our results, WL highlighted as the most informative morphological trait for all species, as WL presented higher differences between species and sexes than AR. A low WL is related to higher energy efficiency to flight and better flight performance [22]. AR shows the flight type of the species, where a high AR shows a higher wing movement speed, which seems to confer the species with a better ability to travel from its natal patches [22, 63, 64]. Accordingly, if both a low WL and a high AR are interpreted as surrogates for increased flight performance, our results show an inconsistency in the case of the Vulnerable *I. sanguinicollis*: this species presented a more energy-efficient flight but a low-speed flight (Fig. 3a, b). However, we believe that this need not be contradictory, as WL and AR measure different traits on flight performance. Therefore, species could have good flight efficiency with respect to body mass but slow flight, characterized by gliding flight according to wing morphology. Based on WL, *I. sanguinicollis* had a higher flight performance than the other species, despite being listed as Vulnerable on the IUCN red list. Hagge et al. [28] also found several good dispersal species among the European red-listed saproxylic beetles. This could be related to the threat posed, even to good dispersers, by their dependence on widely dispersed resources [28]. In contrast to *I. sanguinicollis*, in the also Vulnerable *P. mirifica,* both characters (WL and AR) move the same way, with a low flapping flight combined with a low efficiency flight—this latter character is shared by the other cetonid species (Fig. 3a, b). We can therefore confirm that *P. mirifica* is a poor disperser, consistent with the relict distribution of this species—with 41 localities with Mediterranean distribution (some of them probably already extinct) [47, 74, 75]. As an obligate saproxylic species, this low flight performance together with the regression of their habitats and microhabitats (tree hollows) [6, 16, 76] could seriously jeopardize their populations in the near future. Similarly, the other cetonid species, such as *C. c. aurataeformis* and *P. cuprea,* may also be threatened by habitat loss and connectivity (related to habitat fragmentation) due to their high WL values (Fig. 3a, b). These results are of particular importance, as habitat fragmentation often results in increased autocorrelated extinction patterns that change the cost–benefit balance and lead to less successful emigration overall and increased long-distance dispersal [77].WL and AR revealed differences in flight performance between sexes for some species, such as *E. ferrugineus*, *P. cuprea*, *I. sanguinicollis* and *S. trisignata*; males always had higher flight performance than females (Fig. 4). However, is flight performance a mirror of the dispersal behaviour? The analysis of the differences in the frequency of capture of females and males among traps showed that in *E. ferrugineus,* males would be (1) the sex that travels longer distances and (2) more active in flight than females (Fig. 4), thus having a higher probability of being captured in interception traps (WTs) than in hollow emergence traps (HETs) (Table 4). Accordingly, *E. ferrugineus* dispersal behaviour could be explained by flight performance (the sex with higher flight performance is also the most frequently captured in interception traps). Our results for *E. ferrugineus* are in agreement with the results obtained by Zauli et al. [27], where males were observed to cover greater distances than females. In contrast, the flight performance of *C. c. aurataeformis* did not differ between sexes; thus, the higher capture frequency of females in the WTs was better explained by differences in dispersal behaviour than by the flight performance of females (Fig. 4). Notice that *Cetonia* adults, unlike *E. ferrugineus*, are not saproxylic, and only females search for tree hollows to oviposit. In this way, the postreproductive behaviour of cetonid species, where females lead their dispersal movement towards oviposition sites such as tree hollows and males disperse towards flowers for feed, leaving the breeding sites, reduces the probability of male capture in WTs with respect to females [33, 78]. This supports the theory that females and males may differ in the type of resources they exploit and therefore the impact of resource constraints may vary between the two sexes, limiting their reproductive success, also producing a bias in the behaviour of each sex [79].

### Species sex ratio and sex-biased dispersal

SBD is expected in populations with a high sex ratio bias. Theoretical predictions say that the most abundant sex in natal patches undergoing intense competition would be the most dispersive sex [5, 8, 11, 12, 79]. However, we found a correlation between a female-biased primary sex ratio and low female dispersal ability based on WL (Figs. 4 and 5). This result could be explained by the following: (1) females may have higher dispersal costs than costs due to competition, (2) intrasexual competition is not a decisive factor to induce dispersal bias, or (3) intrasexual competition for oviposition sites or feeding resources (pollen or run sap) simply does not exist within females [12, 62]. Similarly, we know that competition within the saproxylophagous guild that inhabit tree hollows does not seem to be a decisive factor, at least at the interspecific level [80]. Field studies with mark–release–recapture techniques could provide complementary insights into our findings about species dispersal behaviour [1, 81, 82]. Other factors should also be considered to test these theoretical predictions, as the potential benefits of dispersal could overcome physiological costs [12], which could result in an effective dispersal bias between sexes.

## Conclusions

Our results shed light on the value of including a sex perspective in studies related to insect dispersal. Furthermore, the exploration of the possible causes of SBD is useful to better predict the extinction risk of species, as populations of species with poorly dispersive females are more vulnerable to extinction due to their involvement in population dynamics [13]. Based on our results, we stress that SBD is not necessarily driven by intrasexual competition within the most abundant sex. Accordingly, a coevolution between the sex ratio of populations and bias in sex flight performance in saproxylic insects may not always be assumed.

## Data Availability

The datasets used and/or analysed during the current study are available from the corresponding author.

## Abbreviations

SBD: Sex bias dispersal
LRC: Local resource competition
LMC: Local mate competition
IH: Inbreeding hypothesis
WL: Wing loading
AR: Wing aspect ratio
WT: Window trap
HET: Hollow emergence trap
WL_F: Females wing loading
WL_M: Males wing loading
AR_F: Females wing aspect ratio
AR_M: Males wing aspect ratio

## Footnotes

1 Trunk and mould cavities are the main saproxylic microhabitat in veteran trees [16]

## References

[1] Feldhaar H, Schauer B, Ulyshen MD (2018). Dispersal of Saproxylic Insects. Saproxylic insects: diversity, ecology and conservation.

[2] Lawson Handley LJ, Perrin N (2007). Advances in our understanding of mammalian sex-biased dispersal. Mol Ecol.

[3] Shaw AK, Kokko H (2014). Mate finding, Allee effects and selection for sex-biased dispersal. J Anim Ecol.

[4] Asplen MK (2018). Dispersal strategies in terrestrial insects. Curr Opin Insect Sci.

[5] Li XY, Kokko H (2019). Sex-biased dispersal: a review of the theory. Biol Rev.

[6] Baguette M, Benton TG, Bullock JM (2012). Dispersal ecology and evolution.

[7] Trochet A, Courtois EA, Stevens VM, Baguette M, Chaine A, Schmeller DS (2016). Evolution of sex-biased dispersal. Q Rev Biol.

[8] Greenwood PJ (1980). Mating systems, philopatry and dispersal in birds and mammals. Anim Behav.

[9] Dobson FS (1982). Competition for mates and predominant juvenile male dispersal in mammals. Anim Behav.

[10] Pusey AE (1987). Sex-biased dispersal and inbreeding avoidance in birds and mammals. Trends Ecol Evol.

[11] Perrin N, Mazalov V (2000). Local competition, inbreeding, and the evolution of sex-biased dispersal. Amer Naturalist.

[12] Baines CB, Ferzoco IM, McCauley SJ (2017). Sex-biased dispersal is independent of sex ratio in a semiaquatic insect. Behav Ecol Sociobiol.

[13] Prugnolle F, de Meeus T (2002). Inferring sex-biased dispersal from population genetic tools: a review. Heredity.

[14] Turlure C, Baguette M, Stevens VM, Maes D (2011). Species and sex-specific adjustments of movement behavior to landscape heterogeneity in butterflies. Behav Ecol.

[15] Hovestadt T, Mitesser O, Poethke HJ (2014). Gender-specific emigration decisions sensitive to local male and female density. Am Nat.

[16] Micó E, Ulyshen MD (2018). Saproxylic insects in tree hollows. Saproxylic insects: diversity, ecology and conservation.

[17] Müller J, Jarzabek-Müller A, Bussler H, Gossner MM (2014). Hollow beech trees identified as keystone structures for saproxylic beetles by analyses of functional and phylogenetic diversity. Anim Conserv.

[18] Hernández-Corral J, García López A, Ferrández MÁ, Micó E (2021). Physical and biotic factors driving the diversity of spider assemblages in tree hollows of Mediterranean *Quercus* forests. Insect Conserv Diversity.

[19] Warakai D, Okena DS, Igag P, Opiang M, Mack AL (2013). Tree cavity-using wildlife and the potential of artificial nest boxes for wildlife management in New Guinea. Trop Conserv Sci.

[20] Sebek P, Altman J, Platek M, Cizek L (2013). Is active management the key to the conservation of saproxylic biodiversity? Pollarding promotes the formation of tree hollows. PLoS ONE.

[21] Ranius T, Hedin J (2001). The dispersal rate of a beetle, *Osmoderma eremita*, living in tree hollows. Oecologia.

[22] Gibb H, Hjältén J, Ball JP, Pettersson RB, Landin J, Alvini O (2006). Wing loading and habitat selection in forest beetles: are red-listed species poorer dispersers or more habitat-specific than common congenerics?. Biol Conserv.

[23] Nieto A, Alexander KNA (2010). European red list of saproxylic beetles.

[24] Rhodes CJ (2019). Are insect species imperilled? Critical factors and prevailing evidence for a potential global loss of the entomofauna: a current commentary. Sci Prog.

[25] Dubois GF, Le Gouar PJ, Delettre YR, Brustel H, Vernon P (2010). Sex-biased and body condition dependent dispersal capacity in the endangered saproxylic beetle *Osmoderma eremita* (Coleoptera: Cetoniidae). J Insect Conserv.

[26] Lancaster J, Downes BJ (2017). Dispersal traits may reflect dispersal distances, but dispersers may not connect populations demographically. Oecologia.

[27] Zauli A, Chiari S, Hedenström E, Svensson GP, Carpaneto GM (2014). Using odour traps for population monitoring and dispersal analysis of the threatened saproxylic beetles *Osmoderma eremita* and *Elater ferrugineus* in central Italy. J Insect Conserv.

[28] Hagge J, Müller J, Birkemoe T, Buse J, Christensen RHB, Gossner MM (2021). What does a threatened saproxylic beetle look like? Modelling extinction risk using a new morphological trait database. J Anim Ecol.

[29] Bouget C, Brin A, Tellez D, Archaux F (2015). Intraspecific variations in dispersal ability of saproxylic beetles in fragmented forest patches. Oecologia.

[30] Berwaerts K, Dyck HV, Aerts P (2002). Does flight morphology relate to flight performance? An experimental test with the butterfly *Pararge aegeria*. Funct Ecol.

[31] Davis AK, Holden MT (2015). Measuring intraspecific variation in flight-related morphology of monarch butterflies (*Danaus plexippus*): which sex has the best flying gear?. J Insects.

[32] Renault D (2020). A review of the phenotypic traits associated with insect dispersal polymorphism, and experimental designs for sorting out resident and disperser phenotypes. Insects.

[33] Martínez-Pérez S, Sanchez-Rojas G, Galante E, Micó E (2020). Saproxylic cetoniidae (Coleoptera: Scarabaeoidea): a ‘Females’ World’ or a question of dependence on deadwood?. Environ Entomol.

[34] Bouget C, Brustel H, Brin A, Noblecourt T (2008). Sampling saproxylic beetles with window flight traps: methodological insights. Rev Ecol.

[35] Quinto J, Marcos-García MDLÁ, Brustel H, Galante E, Micó E (2013). Effectiveness of three sampling methods to survey saproxylic beetle assemblages in Mediterranean woodland. J Insect Conserv.

[36] Økland B (1996). A comparison of three methods of trapping saproxylic beetles. Eur J Entomol.

[37] Ranius T, Jansson N (2002). A comparison of three methods to survey saproxylic beetles in hollow oaks. Biodivers Conserv.

[38] Hyvarinen E, Kouki J, Martikainen P (2006). A comparison of three trapping methods used to survey forest-dwelling Coleoptera. Eur J Entomol.

[39] Gouix N, Brustel H (2012). Emergence trap, a new method to survey *Limoniscus violaceus* (Coleoptera: Elateridae) from hollow trees. Biodivers Conserv.

[40] Quinto J, Marcos-García MÁ, Díaz-Castelazo C, Rico-Gray V, Brustel H, Galante E (2012). Breaking down complex saproxylic communities: understanding sub-networks structure and implications to network robustness. PLoS ONE.

[41] Micó E, Martínez-Pérez S, Jordán-Núñez J, Galante E, Micó-Vicent B (2022). On how the abandonment of traditional forest management practices could reduce saproxylic diversity in the Mediterranean Region. For Ecol Manag.

[42] Micó E, Galante E. Atlas fotográfico de los escarabeidos florícolas ibero-baleares. Argania editio; 2002.

[43] Micó E, Juárez M, Sánchez A, Galante E (2011). Action of the saproxylic scarab larva *Cetonia aurataeformis* (Coleoptera: Scarabaeoidea: Cetoniidae) on woody substrates. J Nat Hist.

[44] Micó E (2001). Los escarabeidos antófilos de la Península Ibérica (Col. Scarabaeoidea: hopliinae, rutelidae, cetoniidae): taxonomía, filogenia y biología.

[45] Buse J, Ranius T, Assmann T (2008). An endangered longhorn beetle associated with old oaks and its possible role as an ecosystem engineer. Conserv Biol.

[46] Sánchez-Galván IR, Quinto J, Micó E, Galante E, Marcos-García MA (2014). Facilitation among saproxylic insects inhabiting tree hollows in a mediterranean forest: the case of Cetonids (Coleoptera: Cetoniidae) and Syrphids (Diptera: Syrphidae). Environ Entomol.

[47] Verdú JR, Numa C, Galante E. Atlas y libro rojo de los invertebrados amenazados de España (especies vulnerables). Dirección General de Medio Natural y Política Forestal, Ministerio de Medio Ambiente, Medio Rural y Marino; Madrid. 2011.

[48] Verdú JR, Galante E. Libro rojo de los invertebrados de España. Conservación de los insectos saproxílicos del bosque mediterráneo. Dirección General para la Biodiversidad. Ministerio de Medio Ambiente, Madrid; 2006.

[49] García N, Numa C, Bartolozzi L, Brustel H, Buse J, Norbiato M (2019). The conservation status and distribution of Mediterranean saproxylic beetles.

[50] Laibner S (2000). Elateridae of the Czech and Slovak Republics.

[51] Oleksa A, Chybicki IJ, Larsson MC, Svensson GP, Gawroński R (2015). Rural avenues as dispersal corridors for the vulnerable saproxylic beetle *Elater ferrugineus* in a fragmented agricultural landscape. J Insect Conserv.

[52] Tolasch T, von Fragstein M, Steidle JL (2007). Sex pheromone of *Elater ferrugineus* L. (Coleoptera: Elateridae). J Chem Ecol.

[53] Andersson K, Bergman KO, Andersson F, Hedenström E, Jansson N, Burman J (2014). High-accuracy sampling of saproxylic diversity indicators at regional scales with pheromones: the case of *Elater ferrugineus* (Coleoptera, Elateridae). Biol Conserv.

[54] Mannerkoski I, Hyvärinen E, Alexander K, Büche B, Mico E, Pettersson R. Ischnodes sanguinicollis. In: The IUCN Red List of Threatened Species*.* 2010. https://www.iucnredlist.org/species/157829/5154793#assessment-information. Accessed 24 May 2022.

[55] Laz B, Özdikmen H. A new host plant of *Cerambyx (Cerambyx) welensii* (Küster, 1845) (Cerambycidae: Cerambycinae). Munis Entomol Zool 2022;17:1506–12.

[56] Micó E, García-López A, Sánchez A, Juárez M, Galante E (2015). What can physical, biotic and chemical features of a tree hollow tell us about their associated diversity?. J Insect Conserv.

[57] Torres-Vila LM, Mendiola-Díaz FJ, Sánchez-González Á (2018). Adult size and sex ratio variation of *Cerambyx welensii* (Coleoptera: Cerambycidae) in Mediterranean oak (Fagaceae) woodlands. Can Entomol.

[58] Verdugo AV (2011). A propósito de un caso de esquistomelia ternaria heterodinámica de antena derecha en *Stictoleptura trisignata* (Fairmaire, 1852) (Coleoptera: Cerambycidae). Bol Soc Andal Entomol.

[59] Vives E (2000). Coleoptera: Cerambycidae.

[60] Dodelin B, Alexander K, Audisio P, Jansson N, Legakis A, Liberto A, et al. *Stictoleptura trisignata*. In: *The IUCN Red List of Threatened Species.* 2017. https://www.iucnredlist.org/species/86859427/87311762. Accessed 16 May 2022.

[61] Vogel S (2020). Life in moving fluids: the physical biology of flow-revised and expanded.

[62] Angelo MJ, Slansky F (1984). Body building by insects: trade-offs in resource allocation with particular reference to migratory species. Fla Entomol.

[63] Norberg UM (1990). Vertebrate flight.

[64] Malmqvist B (2000). How does wing length relate to distribution patterns of stoneflies (Plecoptera) and mayflies (Ephemeroptera)?. Biol Conserv.

[65] Cousins SAO, Lindborg R, Mattsson S (2009). Land use history and site location are more important for grassland species richness than local soil properties. Nord J Bot.

[66] Vandewoestijne S, Van Dyck H (2011). Flight morphology along a latitudinal gradient in a butterfly: do geographic clines differ between agricultural and woodland landscapes?. Ecography.

[67] Mishra A, Tung S, Shreenidhi PM, Aamir Sadiq M, Shree Sruti VR, Chakraborty PP, Dey S (2018). Sex differences in dispersal syndrome are modulated by environment and evolution. Proc Royal Soc B.

[68] Shapiro SS, Wilk MB (1965). An analysis of variance test for normality (complete samples). Biometrika.

[69] Gotelli NJ, Ellison AM (2004). Primer of ecological statistics.

[70] Zelen M (1971). The analysis of several 2×2 contingency tables. Biometrika.

[71] Beasley TM, Schumacker RE (1995). Multiple regression approach to analyzing contingency tables: post hoc and planned comparison procedures. J Exp Educ.

[72] Sharpe DE (2015). Your Chi-square test is statistically significant: now what?. Pract Assess Res Eval.

[73] Franc N, Götmark F, Økland B, Nordén B, Paltto H (2007). Factors and scales potentially important for saproxylic beetles in temperate mixed oak forest. Biol Conserv.

[74] Tassi F, Aberlenc HP, Rasplus JY, Curletti G, Dutto M, Genson G (2004). Eupotosia mirifica, la grande cétoinebleue, joyaumenacé du patrimoine natural européen. Propositionpour la protection de l’espèce et de sesbiotopes. (Coleoptera Cetoniidae Cetoniinae). Lambillionea.

[75] Agoiz-Bustamante JL, Caselles AB (2009). Sobre la presencia de *Protaetia (Eupotosia) mirifica* (Mulsant, 1842) (Coleoptera: Scarabaeoidea: Cetoniidae) en la Comunidad Autónoma de Extremadura (España). Heteropterus Rev Entomol.

[76] Kirby KJ, Watkins C (1998). Ecological history of European forests.

[77] Fronhofer EA, Stelz JM, Lutz E, Poethke HJ, Bonte D (2014). Spatially correlated extinctions select for less emigration but larger dispersal distances in the spider mite *Tetranychus urticae*. Evolution.

[78] Trizzino M, Bisi F, Morelli CE, Preatoni DG, Wauters LA, Martinoli A (2014). Spatial niche partitioning of two saproxylic sibling species (Coleoptera, Cetoniidae, genus Gnorimus). Insect Conserv Divers.

[79] Li XY, Kokko H (2019). Intersexual resource competition and the evolution of sex-biased dispersal. Front Ecol Evol.

[80] Micó E, Ramilo P, Thorn S, Müller J, Galante E, Carmona CP (2020). Contrasting functional structure of saproxylic beetle assemblages associated to different microhabitats. Sci Rep.

[81] Lowe WH, Allendorf FW (2010). What can genetics tell us about population connectivity?. Mol Ecol.

[82] Ranius T (2006). Measuring the dispersal of saproxylic insects: a key characteristic for their conservation. Popul Ecol.

